# Transformation of Aquatic Plant Diversity in an Environmentally Sensitive Area, the Lake Taihu Drainage Basin

**DOI:** 10.3389/fpls.2020.513788

**Published:** 2020-11-12

**Authors:** Xiaolong Huang, Xuan Xu, Baohua Guan, Shuailing Liu, Hongmin Xie, Qisheng Li, Kuanyi Li

**Affiliations:** ^1^State Key Laboratory of Lake Science and Environment, Nanjing Institute of Geography and Limnology, Chinese Academy of Sciences, Nanjing, China; ^2^Jiangsu JiangDa Eco Technology Co., Ltd., Wuxi, China; ^3^Sino-Danish College, University of Chinese Academy of Sciences, Beijing, China; ^4^College of Environmental and Chemical Engineering, Chongqing Three Gorges University, Wanzhou, China

**Keywords:** Yangtze River, aquatic plant, biodiversity index, Lake Taihu drainage basin, *Eichhornia crassipes*, *Cabomba caroliniana*

## Abstract

Located downstream of the Yangtze River Delta, the Lake Taihu drainage basin (LTDB) is one of the most developed areas in China. This area currently faces population and development issues, as well as many environmental problems, such as cultural eutrophication, algal blooms, and loss of native aquatic plants. Changes in aquatic biodiversity have received less attention than have changes in terrestrial habitats because relevant observations are lacking. In this study, information from 2010, 2014, and 2018 concerning the transformation of the aquatic plant biodiversity was obtained. The results showed that the dominant aquatic plants have changed from native plants to invasive plants. Aquatic plant biodiversity showed a decreasing trend, which may reduce the freshwater ecosystem function, and anthropogenic activities accounted for these changes. How to prevent the decline in aquatic plants and control the invasion of introduced aquatic plants should be a priority in the management of aquatic plants in the LTDB.

## Introduction

Aquatic ecosystems provide irreplaceable economic and cultural services to human societies and are currently experiencing more significant loss compared to terrestrial ecosystems (Dudgeon et al., 2006). Most shallow lakes (<7 m) with water turnover rates of less than one year are essential components of the freshwater ecosystem (Ji, 2008). Currently, lakes in China are facing a series of ecological and environmental problems, such as water area loss, the fragmentation of lake ecosystems, a decline in biodiversity, and the weakening of ecological functions. Anthropogenic influences (including water pollution, diking, draining, and conversion to agricultural or urban use) have caused the degradation of many shallow lakes (Fang et al., 2006; An et al., 2013). Lake Taihu has been viewed as a model shallow lake in China (Qin, 2008; Hu, 2016). Many articles on the transition and management of aquatic plants in this lake [e.g., Zhang et al. (2018), and Wang et al. (2019)] have been published. However, direct evidence of the transformation of dominant aquatic plants and aquatic plant diversity in the Lake Taihu drainage basin (LTDB) is rare because of the lack of long-term field observations. Located downstream of the Yangtze River Delta, which is one of the most developed and populated areas in China, the Taihu Basin occupies only 1% (36,900 km^2^) of the total territorial area but encompassed 4.4% (60.58 million) of the total population and contributed 9.8% [8,081.5 billion renminbi (RMB, the official currency of China)] of the gross domestic product (GDP) of China in 2017 (Taihu Basin Authority TBA, 2017; Zhang et al., 2018). A serious eutrophication trend has been detected in this area, and some lakes have transformed from being dominated by submerged vegetation to being dominated by algae, indicating this area is an environmentally sensitive area (Mitsch and Gosselink, 2011; Zhang et al., 2017; Wang et al., 2019). Moreover, studies on invasive aquatic plants have received little attention, although many field investigations on aquatic plants have been conducted in this area. Remote sensing has been widely used for the recognition of aquatic plants, but distinguishing distinct aquatic plants *via* this method is challenging (Liu et al., 2015). Traditional field investigations are thus still indispensable.

As primary producers of trophic chains, aquatic plants provide food and shelter for fishes. Additionally, aquatic plants provide breeding grounds for benthic organisms (Bornette and Puijalon, 2011). Losses and declines in native aquatic vegetation in the LTDB have prompted extensive concerns (Gao et al., 2017; Zhang et al., 2017). Owing to the flourishment of aquarium markets, many introduced ornamental plants are imported and sold in China without undergoing environmental impact assessments, and these activities also contribute to the spread of introduced plants (Xu et al., 2006; Jiang et al., 2011). However, synchronal *in situ* studies on both native and introduced invasive species have received little attention in this area. On the basis of previous investigations, the main invasive plants in Lake Taihu are water hyacinth (*Eichhornia crassipes*) and alligator weed (*Alternanthera philoxeroides*). A new invasive plant, Carolina fanwort (*Cabomba caroliniana*), has recently received considerable amounts of attention. An introduced plant species, but one not listed as an invasive plant, called parrot’s feather (*Myriophyllum aquaticum*) was also found in the LTDB.

In the Intergovernmental Science-Policy Platform on Biodiversity and Ecosystem Services *Global Assessment Report*, invasive species were identified as one of the top five global factors driving negative changes in natural habitats around the world. Although biological invasion is a natural process, it is intensified by human activities. Especially in modern times, biological invasion has become more frequent and complicated due to international trade, horticulture, immigration, and other cultural and commercial exchanges (Nentwig, 2008a). Economic damage caused by invasive species accounts for approximately 5% of the world’s gross national product (GNP) (Pimentel et al., 2008). In recent years, biological invasions have become increasingly severe in China (Feng and Zhu, 2010; Wu and Ding, 2019). As the world’s largest import and export country, China’s trade activities have increased considerably since the 1980s (WTO, 2017). The trade link between China and the world is becoming tighter than ever before, and trade can introduce commercial, ornamental, and non-native species accidentally or deliberately (Nentwig, 2008b). Coupled with the high diversity of China’s ecosystems, it is more conducive to the invasion and spread of introduced species, which also renders China one of the most seriously endangered places.

In this study, the biodiversity and distributions of native and introduced aquatic plants in the LTDB *in situ* were obtained through field investigations in 2010, 2014, and 2018. We aimed to address the *status quo* of invasive aquatic plants in the LTDB and to help develop reasonable means to control invasive plants in China. Three issues are discussed: (1) the identification of aquatic plants with a dominant role in the LTDB; (2) the relationships among aquatic plant biodiversity indices, the human population, and the economy; and (3) possible control methods for invasive plants in the LTDB.

## Materials and Methods

### Study Area

The area of the LTDB includes Suzhou, Wuxi, and Changzhou in Jiangsu Province; Jiaxing, Huzhou, and Hangzhou in Zhejiang Province; and the mainland Shanghai Municipality (Qin et al., 2007; Zhang et al., 2018). The main lakes in the LTDB include Lake Taihu, Lake Gehu, and Lake Yangchenghu.

### Data Collection

**FIGURE 1:**
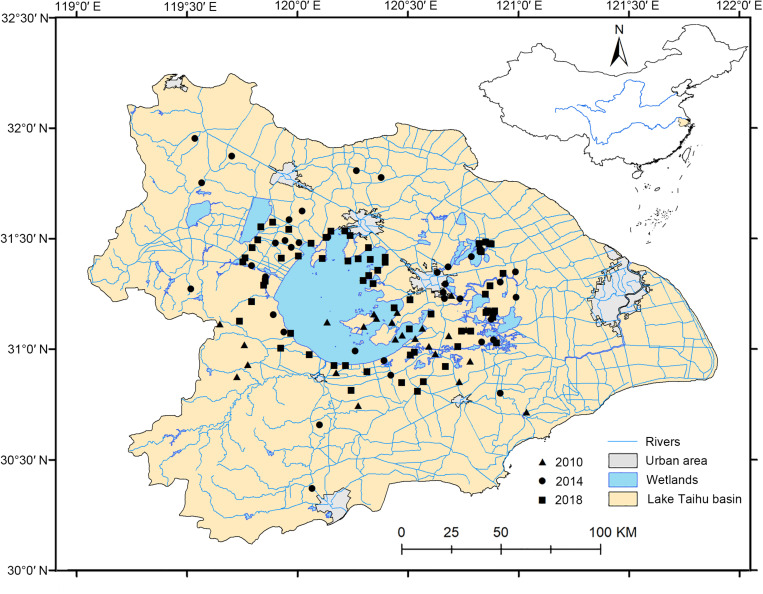
Distributions of aquatic plant quadrats in the Lake Taihu drainage basin (LTDB) in 2010, 2014, and 2018.

Field investigations were performed in the LTDB in July 2010, August 2014, and from June to October 2018. We did not use snorkels or scubas or other professional diving equipment during our investigation. Fortunately, the ponds, lakes, channels, or rivers in our investigations are not deep. For example, the average depth of the largest lake in the LTDB, Lake Taihu, is only 1.9 m, allowing us to use free-diving to conduct our investigation without rakes to minimize sampling errors. The sampling quadrats were randomly selected, with an area of 1 × 1 m^2^, and were approximately 5 km apart (Figure 1). Handheld GPS recorders were used to capture location coordinates (longitudes, latitudes, and elevations) in the field investigations. Location information, water properties, habitat types, and surrounding terrestrial vegetation types were recorded. Plant specimens were collected, classified, and then identified according to the *Flora of China*. The number of each dominant and companion plant species was counted. Water quality indicators including the pH, conductivity, dissolved oxygen, and salinity were determined by a YSI multiparameter water quality analyzer (YSI Inc., Yellow Springs, OH, United States). The sample information is listed in the Supplementary Material.

The relative coverage (RC) and relative abundance (RA) of each species in a sampling plot were measured. The RC was estimated by visual assessment, and a species’ RC = projective coverage/all projective coverage, and a species’ RA = number of a species/numbers of all species in a sampling plot. The RC and RA of a species were calculated in every individual sampling plot, and their sums were used to calculate the relative importance value (RIV). The species’ relative frequency (RF) was calculated as RF = number of a species/number of all plants in all sampling plots of a specific year. The RIV of a species, that is, dominant advantage species index, is the sum of the species’ RCs and RAs in all sampling plots and RF of a specific year according to the following formula (Jing et al., 2014; Wu et al., 2017):

*RIV* = (Σ*RC* + Σ*RA* + *RF*)/3

Three α-species aquatic plant diversity indices of the plots were calculated according to the following formulae (Ricklefs, 2008; Colwell, 2009; Molles, 2015):

Simpson diversity index: *D* = 1 −Σ*P*_*i*_^2^ = 1 −Σ(*N*_*i*_/*N*)^2^

Shannon-Wiener diversity index: *H* = −Σ*P*_*i*_ ln *P*_*i*_

Species evenness (Pielou) index: *E* = (−Σ*P*_*i*_ ln *P*_*i*_)/ln *N*

where *P_*i*_* = *N*_*i*_/*N*, *N*_*i*_ is the number of a particular plant species in a plant sampling plot, and *N* is the total number of all plant species in a plant sampling plot.

The population and GDP data from the years 2008 to 2017 were obtained from the Taihu Basin Authority of the Ministry of Water Resources, China^1^. As the data from the year 2018 were not yet released, they were calculated according to the sums of the population and GDP data in this area.

### Data Analysis

The data were tested to meet the assumptions of a normal distribution and homoscedasticity of variances before the statistical analysis, and these assumptions were verified by the Shapiro–Wilk test and Levene’s test, respectively. A one-way analysis of variance was used to examine differences (*P* = 0.05) in plant biodiversity indices among the years 2010, 2014, and 2018. Simple linear regression models were constructed to analyze the relationships among the year, plant biodiversity indices, population, and GDP. Multiple regression models were constructed to analyze the relationships among plant biodiversity indices and the coverage of two invasive plants, *E. crassipes* and *Ca. caroliniana*. Statistical analyses were conducted in SPSS Statistics 19 (IBM Corp., Armonk, NY, United States). Mapping was performed in ArcGIS 10.3 (Esri Corp., Redlands, CA, United States).

## Results

### Transformations of Dominant Aquatic Plants in the LTDB

Twenty-four field quadrats were obtained in July 2010, among which 34 aquatic plants were collected in total. The native plant *Ceratophyllum demersum* had the highest RIV (RIV = 10.68), followed by the invasive plant *E. crassipes* (RIV = 9.59) (Table 1).

**TABLE 1:** The relative importance value (RIV) of aquatic plant species in the Lake Taihu drainage basin (LTDB) in 2010.

**Species**	**Status**	***RIV***	**Species**	**Status**	***RIV***
*Ceratophyllum demersum*	Native	10.68	*Nymphoides indica*	Native	1.23
*Eichhornia crassipes*	Invasive	9.59	*Najas marina*	Native	1.11
*Myriophyllum spicatum*	Native	8.68	*Marsilea quadrifolia*	Native	1.07
*Hydrilla verticillata*	Native	7.66	*Pistia stratiotes*	Invasive	0.79
*Potamogeton wrightii*	Native	5.77	*Cabomba caroliniana*	Invasive	0.53
*Hydrocharis dubia*	Native	5.19	*Nymphoides peltata*	Native	0.51
*Vallisneria natans*	Native	4.77	*Alternanthera philoxeroides*	Invasive	0.50
*Nelumbo nucifera*	Native	4.36	*Hygroryza aristata*	Native	0.46
*Utricularia vulgaris*	Native	4.34	*Monochoria vaginalis*	Native	0.44
*Lemna minor*	Native	3.80	*Sparganium stoloniferum*	Native	0.15
*Potamogeton crispus*	Native	2.06	*Trapella sinensis*	Native	0.13
*Ludwigia adscendens*	Native	1.91	*Trapa japonica*	Native	0.12
*Salvinia natans*	Native	1.87	*Zizania latifolia*	Native	0.07
*Alisma plantago-aquatica*	Native	1.41	*Phragmites australis*	Native	0.03
*Najas minor*	Native	1.39	*Trapa bicornis*	Native	0.02
*Typha orientalis*	Native	1.37	*Vallisneria denseserrulata*	Native	0.02
*Myriophyllum verticillatum*	Native	1.34	*Sagittaria trifolia* var. *sinensis*	Native	0.02

In total, 36 field quadrats were obtained in August 2014, including 35 aquatic plants. The invasive plant *E. crassipes* replaced *Cer. demersum*, achieving the highest RIV (RIV = 12.44), which was followed by that of *Vallisneria natans* (RIV = 11.59) (Table 2).

**TABLE 2:** The relative importance value (RIV) of aquatic plant species in the Lake Taihu drainage basin (LTDB) in 2014.

**Species**	**Status**	***RIV***	**Species**	**Status**	***RIV***
*Eichhornia crassipes*	Invasive	12.44	*Potamogeton crispus*	Native	2.05
*Vallisneria natans*	Native	11.59	*Myriophyllum verticillatum*	Native	1.93
*Ceratophyllum demersum*	Native	10.73	*Calla palustris*	Native	1.76
*Cabomba caroliniana*	Invasive	9.08	*Ipomoea aquatica*	Native	1.59
*Lemna minor*	Native	9.02	*Euryale ferox*	Native	0.98
*Hydrilla verticillata*	Native	8.46	*Alternanthera philoxeroides*	Invasive	0.88
*Hydrocharis dubia*	Native	7.55	*Phragmites australis*	Native	0.73
*Salvinia natans*	Native	7.51	*Najas minor*	Native	0.71
*Myriophyllum spicatum*	Native	6.45	*Monochoria vaginalis*	Native	0.67
*Nelumbo nucifera*	Native	5.57	*Pistia stratiotes*	Invasive	0.65
*Nymphoides peltate*	Native	5.13	*Blyxa japonica*	Native	0.40
*Potamogeton wrightii*	Native	4.09	*Trapella sinensis*	Native	0.14
*Ludwigia adscendens*	Native	3.98	*Trapa bicornis*	Native	0.07
*Nymphoides indica*	Native	3.85	*Myriophyllum aquaticum*	Introduced	0.02
*Trapa japonica*	Native	3.07	*Vallisneria denseserrulata*	Native	0.02
*Typha orientalis*	Native	2.69	*Sparganium stoloniferum*	Native	0.01
*Zizania latifolia*	Native	2.50	*Sagittaria trifolia* var. *sinensis*	Native	0.01
*Najas marina*	Native	2.41			

In total, 67 field quadrats were obtained from June to October 2018, and a total of 35 aquatic plants were obtained. The invasive plant *Ca. caroliniana* had the highest RIV (RIV = 16.84), which was followed by that of *E. crassipes* (RIV = 13.65) (Table 3).

**TABLE 3:** The relative importance value (RIV) of aquatic plant species in the Lake Taihu drainage basin (LTDB) in 2018.

**Species**	**Status**	***RIV***	**Species**	**Status**	***RIV***
*Cabomba caroliniana*	Invasive	16.84	*Euryale ferox*	Native	1.17
*Eichhornia crassipes*	Invasive	13.65	*Vallisneria natans*	Native	0.91
*Hydrilla verticillata*	Native	9.07	*Nelumbo nucifera*	Native	0.88
*Ceratophyllum demersum*	Native	7.71	*Nymphoides peltata*	Native	0.71
*Lemna minor*	Native	7.21	*Phragmites australis*	Native	0.68
*Hydrocharis dubia*	Native	7.03	*Ipomoea aquatica*	Native	0.67
*Myriophyllum spicatum*	Native	5.15	*Pistia stratiotes*	Invasive	0.60
*Potamogeton wrightii*	Native	4.61	*Najas marina*	Native	0.45
*Alternanthera philoxeroides*	Invasive	4.60	*Stuckenia filiformis*	Native	0.43
*Ludwigia adscendens*	Native	2.54	*Sagittaria trifolia* var. *sinensis*	Native	0.19
*Trapa japonica*	Native	2.50	*Typha orientalis*	Native	0.13
*Trapa bicornis*	Native	2.35	*Utricularia vulgaris*	Native	0.12
*Myriophyllum aquaticum*	Introduced	2.06	*Potamogeton crispus*	Native	0.11
*Vallisneria denseserrulata*	Native	1.81	*Alisma plantago-aquatica*	Native	0.10
*Trapa incisa*	Native	1.80	*Hygroryza aristata*	Native	0.09
*Nymphoides indica*	Native	1.72	*Calla palustris*	Native	0.09
*Salvinia natans*	Native	1.27	*Typha angustifolia*	Native	0.03
*Zizania latifolia*	Native	1.23			

### Relationships of the Aquatic Plant Biodiversity With Population and GDP

**FIGURE 2:**
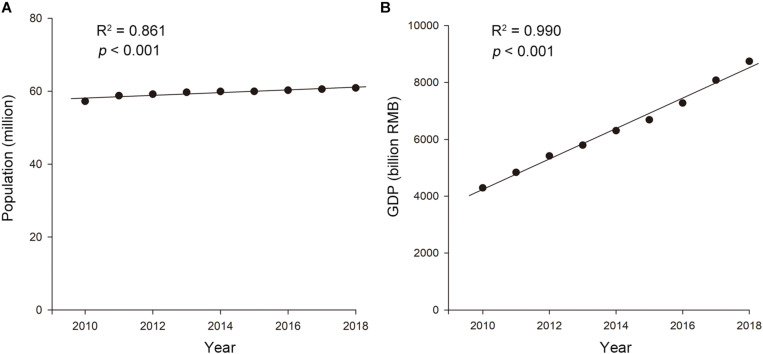
The trends in the **(A)** population and **(B)** GDP in the Lake Taihu drainage basin (LTDB) from 2010 to 2018.

The population of the LTDB experienced slight growth, from 57.24 million in 2010 to 60.91 million in 2018 (*R*^2^ = 0.861, *p* < 0.001; Figure 2A). Additionally, the LTDB experienced rapid GDP growth, from 4290.5 billion RMB (681.1 billion USD) in 2010 to 8746.2 billion RMB (1388.2 billion USD) in 2018 (*R*^2^ = 0.990, *p* < 0.001; Figure 2B), representing a more than a 2-fold increase.

**FIGURE 3:**
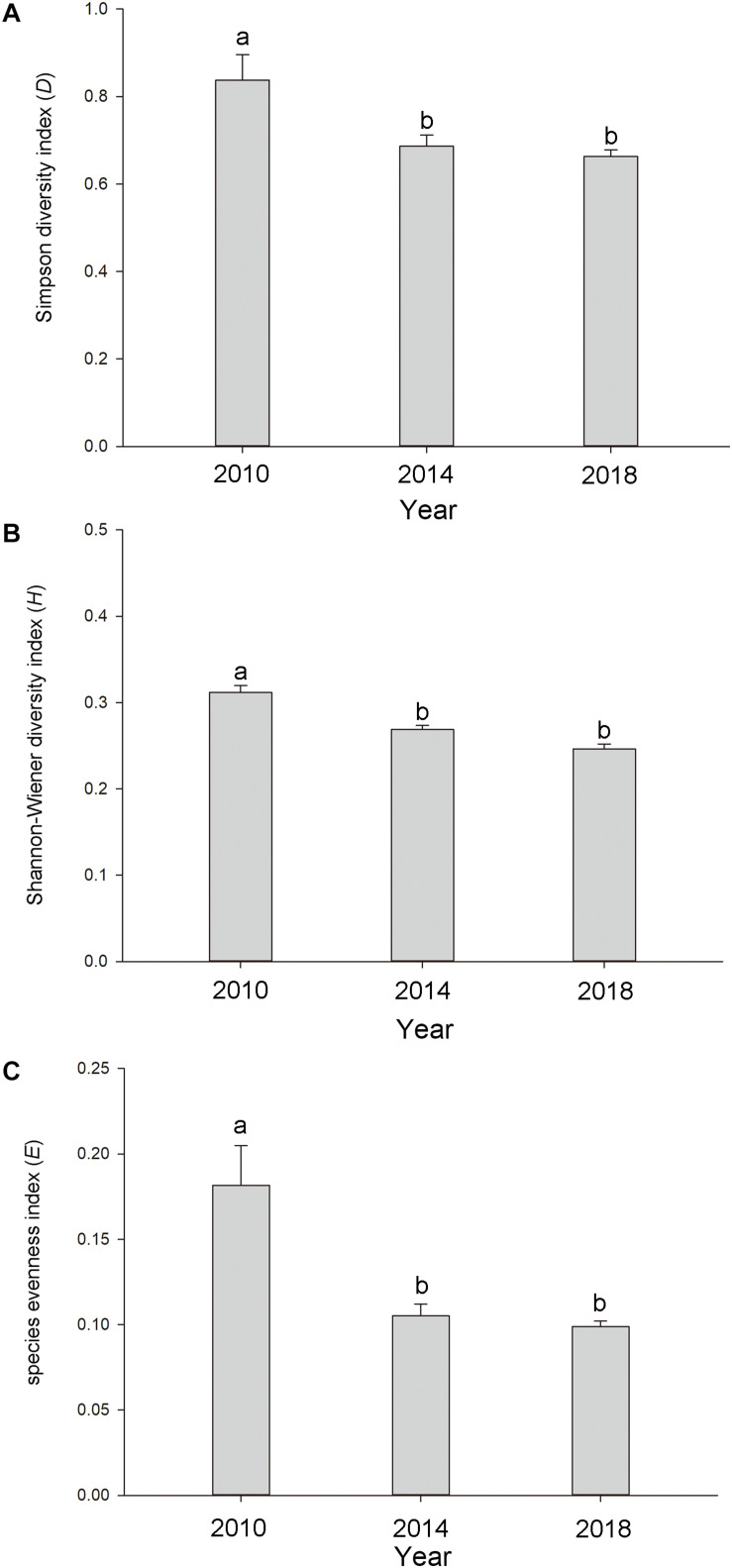
The **(A)** Simpson diversity index, **(B)** Shannon-Wiener diversity index, and **(C)** species evenness index in the Lake Taihu drainage basin (LTDB) in 2010, 2014, and 2018. The values are represented as means ± SE. Different lowercase letters indicate significant differences among the different years (*p* < 0.05).

The three α-biodiversity indices showed similar tendencies and were significantly higher in 2010 (*D* = 0.837 ± 0.058, *H* = 0.328 ± 0.039, and *E* = 0.182 ± 0.023; means ± SE) (*p* < 0.05) than in 2014 (*D* = 0.686 ± 0.026, *H* = 0.269 ± 0.005, and *E* = 0.105 ± 0.007; means ± SE) and 2018 (*D* = 0.663 ± 0.016, *H* = 0.246 ± 0.006, and *E* = 0.099 ± 0.003; means ± SE) (Figure 3). The three α-biodiversity indices did not show dramatic differences between 2014 and 2018 (Figure 3).

Linear regressions of aquatic plant biodiversity with the population and GDP data in the years 2010, 2014, and 2018 were performed to evaluate the relevance between the biodiversity and anthropogenic activities (Tables 4, 5). The results showed significant negative correlations when comparing the three α-species plant diversity indices with the population and GDP data (Tables 4, 5).

**TABLE 4:** The estimated equations and *R*^2^ and *p* values for the Simpson diversity index (*D*), Shannon-Wiener diversity index (*H*), and species evenness index (*E*) in relation to the population (pop.) in the Lake Taihu drainage basin (LTDB).

	**Equation**	***R*^2^**	***p***
Simpson diversity index (*D*)	*D* = 3.654 − 0.049 × pop.	0.982	<0.001
Shannon-Wiener diversity index (*H*)	*H* = 1.311 − 0.017 × pop.	0.993	<0.001
Species evenness index (*E*)	*E* = 1.541 − 0.024 × pop.	0.963	<0.001

**TABLE 5:** The estimated equations and *R*^2^ and *p* values for the Simpson diversity index (*D*), Shannon-Wiener diversity index (*H*), and species evenness index (*E*) in relation to the gross domestic product (GDP) (billion RMB) in the Lake Taihu drainage basin (LTDB).

	**Equation**	***R*^2^**	***p***
Simpson diversity index (*D*)	*D* = 0.974 – 3.807 × GDP	0.858	<0.001
Shannon-Wiener diversity index (*H*)	*H* = 0.369 – 1.452 × GDP	0.947	<0.001
Species evenness index (*E*)	*E* = 0.245 – 1.802 × GDP	0.806	<0.001

### Relationship Between the Coverage of Invasive Plants and Plant Biodiversity in the LTDB

**FIGURE 4:**
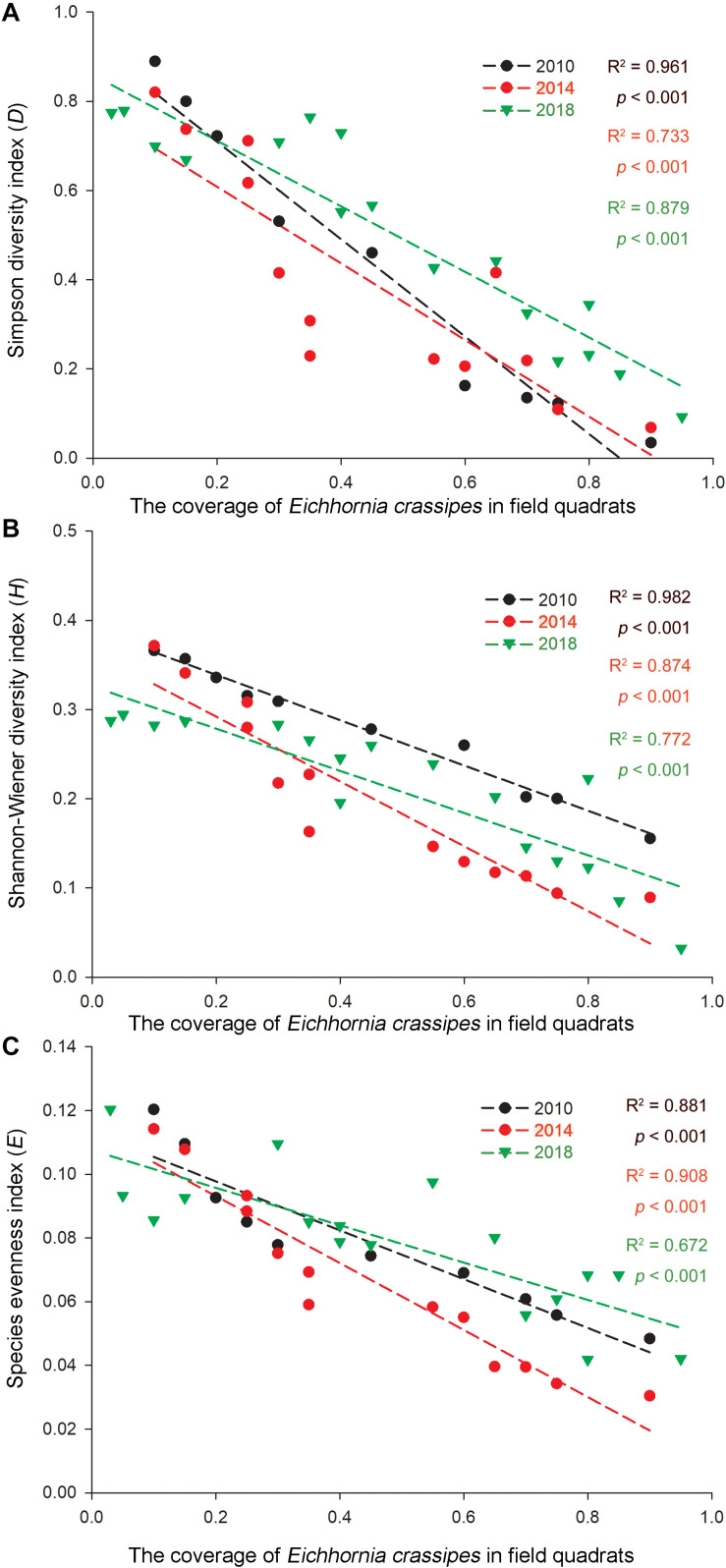
Correlations of the **(A)** Simpson diversity index, **(B)** Shannon-Wiener diversity index, and **(C)** species evenness index with the relative coverage (*RC*) of *E. crassipes* in the Lake Taihu drainage basin (LTDB) in 2010, 2014, and 2018.

**FIGURE 5:**
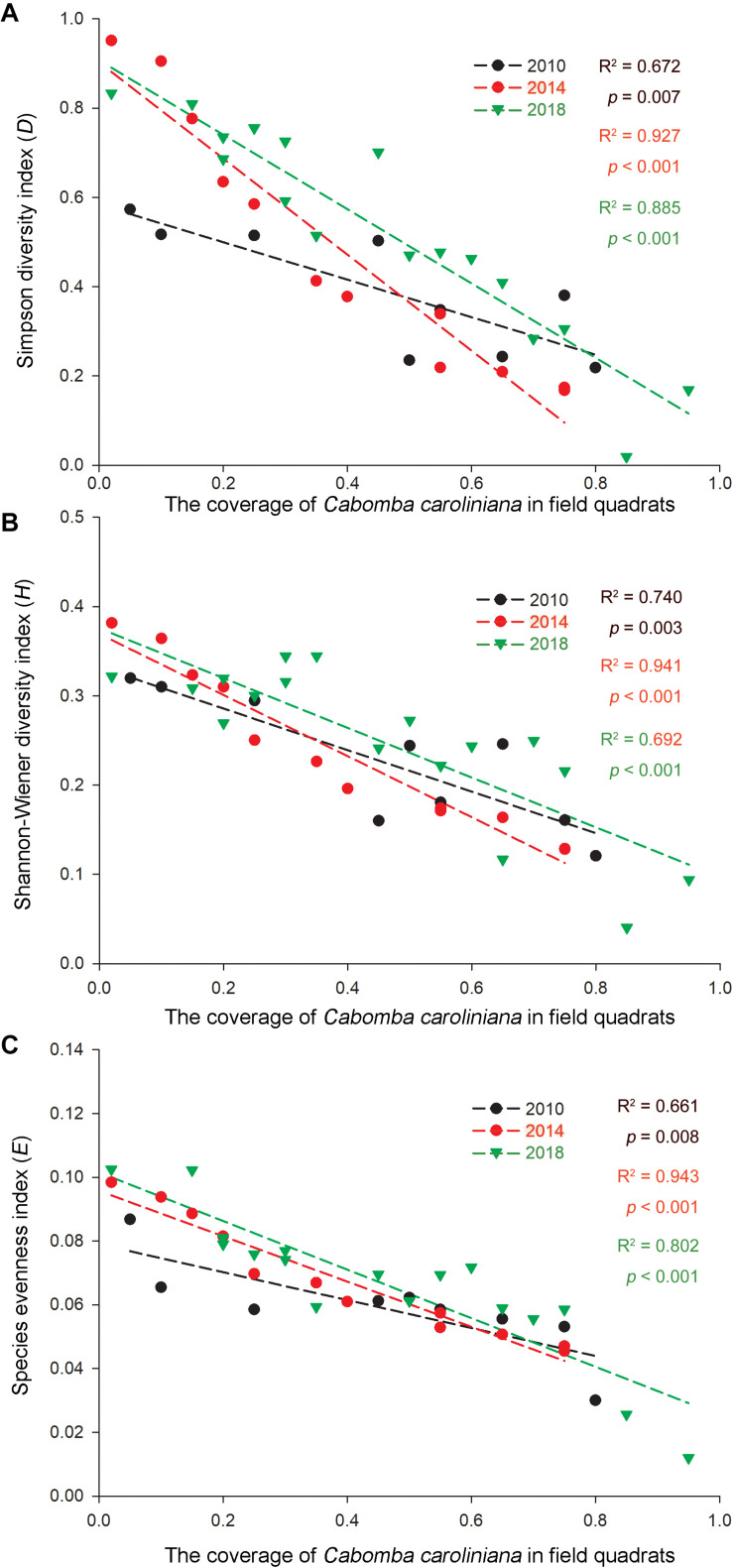
Correlations of the **(A)** Simpson diversity index, **(B)** Shannon-Wiener diversity index, and **(C)** species evenness index with the relative coverage (*RC*) of *Ca. caroliniana* in the Lake Taihu drainage basin (LTDB) in 2010, 2014, and 2018.

As invasive *Ca. caroliniana* and *E. crassipes* had become the dominant aquatic species in the field quadrats in this study (Table 3), the linear regression of the coverage and the three α-species aquatic plant diversity indices of the two species in the aquatic plant sampling plots in 2010, 2014, and 2018 are shown in Figures 4, 5. The results indicate that with increasing coverage of the two plants, a negative trend appeared for the three α-species aquatic plant diversity indices regardless of the different years, and in some sampling plots, an invasive plant sometimes created a mono-species community.

## Discussion

### Transformation of Aquatic Plants and Loss of Aquatic Plant Biodiversity in the LTDB

In this study, we found that the total numbers of aquatic plants in 2010, 2014, and 2018 in the LTDB did not have a significant change, but the aquatic plant vegetation has transformed from being dominated by the native plant *Cer. demersum* to being dominated by the invasive plants *Ca. caroliniana* and *E. crassipes* in the aquatic sampling plots. Aquatic plant biodiversity showed a decreasing trend, which may reduce the freshwater ecosystem function, and anthropogenic activities were responsible for these changes. This transformation may cause declines in native species and the extinction of narrowly distributed species, as invasive species typically have advantageous traits that facilitate their competitiveness with native species, and they are more tolerant of eutrophication and other human pollution, often being able to survive habitat disturbances to become dominant species (Richards et al., 2006; van Kleunen et al., 2010).

A previous study showed a strong relationship between the human population and invasive plant species richness (Weber et al., 2008). Duan et al. (2009) showed that the GDP is the dominant factor in the initial blooming date and that the GDP per capita is the dominant factor for blooming duration in Lake Taihu. Similarly, the population and GDP had negative impacts on the aquatic plant biodiversity in the LTDB in this study (Tables 4, 5). Previous studies have shown that biodiversity loss reduces the ecosystem function (Schnitzer et al., 2011; Huang et al., 2018), and the loss of aquatic plant biodiversity may change the freshwater ecosystem function in the LTDB permanently. Human activities are not well quantified; thus, we are not implicating the population or GDP growth as accounting for these changes. The good linear regressions of the aquatic plant biodiversity with the population and GDP data does not indicate that these characteristics are the exact causes of the two relationships; they are simply proper indicators that may provide awareness of the relationship between the biodiversity and anthropogenic activities.

Remote sensing data indicate that the distribution of aquatic plants in the LTDB showed a gradually increasing trend from 1980 to 2014 and a sharp decrease in 2015, and the distribution remained at a low level until 2017 (Wang et al., 2019). In this study, we found a decreasing trend for the biodiversity of aquatic plants (Figure 3), and the invasive plants *Ca. caroliniana* and *E. crassipes* directly reduced the plant biodiversity (Figures 4, 5), which is consistent with previous studies that found that biological invasion is an essential factor that driving the decrease in the biodiversity of plant communities (Ryser and Eek, 2000; Vilà et al., 2011). High biodiversity typically increases resistance to biological invasions, as high biodiversity affords greater resistance to invasion and limits the availability of vacant niches for new invaders (the “*biotic resistance hypothesis*”) (Mitchell et al., 2006; Fan et al., 2013). The transformation from dominantly native species to dominantly invasive species and the loss of aquatic plant biodiversity may cause irreversible ecosystem shifts in the LTDB.

### Possible Management Implications for Invasive Plants

*Eichhornia crassipes* was introduced into China as an ornamental plant in the early twentieth century and quickly spread after its escape from domestic surroundings (Pan et al., 2012; Wu and Ding, 2019). The plant has a fast growth ability, high sexual and asexual reproductive capacities, a relatively short growth period, and low genetic differentiation, causing the plant to become the most widely distributed invasive aquatic plant worldwide (Zhang et al., 2010; International Union for Conservation of Nature IUCN, 2013). The plant can cover the water surfaces to form a continuous floating mat, which is extremely harmful to aquatic habitats (Charles and Dukes, 2008; Michelan et al., 2018). It is difficult to eradicate from a water body using the salvage method (Patel, 2012). The use of herbicides causes secondary pollution in the water bodies (Feng et al., 2017; Mishra and Maiti, 2017). Biological control methods for *E. crassipes* using insects on have been proven to be successful in Louisiana, United States (Wainger et al., 2018), but a similar biocontrol method has not been applied in the LTDB, and the potential impact of introduced insects on the local environment remains unknown. Bicudo et al. (2007) also showed that removal of *E. crassipes* from a reservoir was inefficient and led to a more turbid state.

*Cabomba caroliniana* is a perennial submerged plant species that is native to the United States and South America and is often introduced as an aquarium plant in the rest of the world (Wilson et al., 2007; McCracken et al., 2013). The plant was introduced into China as an ornamental plant in the 1980s (Ding et al., 2003). Owing to its unique leaf shape and ease of cultivation, it was sold in aquarium markets in southern China and later turned into a weed that was difficult to control. The plant was widely used in the construction of wetland parks and river channel restoration in China due to its high water purification, pollution tolerance, and esthetic values before it was included on the list of invasive species (Zou et al., 2012). Currently, *Ca. caroliniana* is in the early stage of its invasive outbreak in China, as it was only recently recognized as an invasive plant (Ministry of Ecology and Environment MEE, 2016). No extraordinary removal measures have been developed, and salvage requires a high amount of manpower. During this process, the species easily forms stem fragments that may produce new populations with water flow, benefiting its spread (Scheers et al., 2019). The rapid spread of *Ca. caroliniana* may indicate that the plant has not yet reached its distribution limit.

## Conclusion

In this study, the aquatic plant biodiversity in the LTDB was obtained in 2010, 2014, and 2018. The results showed that the aquatic plant dominance has transformed from native plants to the invasive plants *E. crassipes* and *Ca. caroliniana*. The aquatic plant biodiversity has experienced a decreasing trend, which may result in changes to the structure and function of aquatic ecosystems in the LTDB. Additionally, the results of this study indicate that anthropogenic activities may have accounted for these changes.

## Data Availability Statement

All datasets generated for this study are included in the manuscript/Supplementary Material.

## Author Contributions

XH and XX designed and executed the research project. XH, SL, HX, and QL collected the field data. XH, XX, BG, and KL led the statistical analysis and drafted the manuscript. All authors contributed to the article and approved the submitted version.

## Conflict of Interest

The authors declare that the research was conducted in the absence of any commercial or financial relationships that could be construed as a potential conflict of interest.

## Footnotes

1 http://www.tba.gov.cn/
